# Bis[4-(dimethyl­amino)pyridinium] hexa­kis[bromido/chlorido(0.78/0.22)]stannate(IV)

**DOI:** 10.1107/S1600536809019734

**Published:** 2009-06-06

**Authors:** Kong Mun Lo, Seik Weng Ng

**Affiliations:** a Department of Chemistry, University of Malaya, 50603 Kuala Lumpur, Malaysia

## Abstract

The Sn atom in the title salt, (C_7_H_11_N_2_)_2_[SnBr_4.67_Cl_1.33_], lies on a center of symmetry within an octa­hedron of disordered halogen atoms. The three independent halogen atoms are each a mixture of bromine and chlorine atoms [with site occupancies for bromine of 0.614 (1), 0.831 (1) and 0.888 (1)]. An N—H⋯ hydrogen bond is present.

## Related literature

For the isostructural tribromidotrichloridostannate, see: Lo & Ng (2008 ▶); for the isostructural penta­bromido­chlorido­stannate, see: Jang *et al.* (2009 ▶).
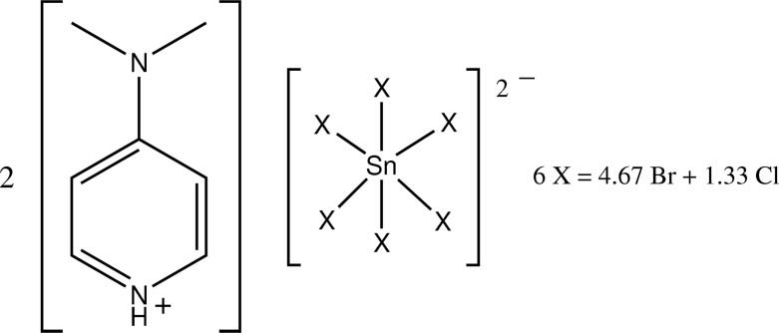


## Experimental

### 

#### Crystal data

(C_7_H_11_N_2_)_2_[SnBr_4.67_Cl_1.33_]
*M* *_r_* = 785.15
Monoclinic, 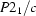

*a* = 8.4530 (2) Å
*b* = 11.9036 (2) Å
*c* = 11.9093 (2) Å
β = 107.109 (1)°
*V* = 1145.30 (4) Å^3^
*Z* = 2
Mo *K*α radiation
μ = 9.42 mm^−1^
*T* = 100 K
0.30 × 0.25 × 0.20 mm

#### Data collection

Bruker SMART APEX diffractometer
Absorption correction: multi-scan (*SADABS*; Sheldrick, 1996 ▶) *T*_min_ = 0.504, *T*_max_ = 0.746 (expected range = 0.103–0.152)
10319 measured reflections
2622 independent reflections
2240 reflections with *I* > 2σ(*I*)
*R*_int_ = 0.033

#### Refinement

*R*[*F* ^2^ > 2σ(*F* ^2^)] = 0.023
*wR*(*F* ^2^) = 0.060
*S* = 0.99
2622 reflections
127 parameters
6 restraints
H atoms treated by a mixture of independent and constrained refinement
Δρ_max_ = 0.80 e Å^−3^
Δρ_min_ = −0.87 e Å^−3^

### 

Data collection: *APEX2* (Bruker, 2007 ▶); cell refinement: *SAINT* (Bruker, 2007 ▶); data reduction: *SAINT*; program(s) used to solve structure: *SHELXS97* (Sheldrick, 2008 ▶); program(s) used to refine structure: *SHELXL97* (Sheldrick, 2008 ▶); molecular graphics: *X-SEED* (Barbour, 2001 ▶); software used to prepare material for publication: *publCIF* (Westrip, 2009 ▶).

## Supplementary Material

Crystal structure: contains datablocks global, I. DOI: 10.1107/S1600536809019734/tk2458sup1.cif

Structure factors: contains datablocks I. DOI: 10.1107/S1600536809019734/tk2458Isup2.hkl

Additional supplementary materials:  crystallographic information; 3D view; checkCIF report

## Figures and Tables

**Table 1:** Hydrogen-bond geometry (Å, °)

*D*—H⋯*A*	*D*—H	H⋯*A*	*D*⋯*A*	*D*—H⋯*A*
N1—H1⋯Br1	0.88 (1)	2.484 (18)	3.334 (3)	162 (4)

**Fig. 1.**
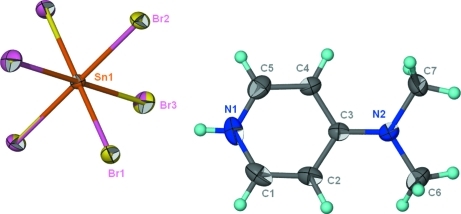
Thermal ellipsoid plot (Barbour, 2001) of 2[C7H11N2]+ [SnBr4.67Cl1.33]2- at the 70% probability level. Hydrogen atoms are drawn as spheres of arbitrary radius. The bromine atoms are disordered with respect to the chlorine atoms.

## supplementary crystallographic information

### Experimental

Dibenzyltin dichloride (0.37 g, 1 mmol) and 4-dimethylaminopyridine hydrobromide perbromide (0.73 g, 2 mmol) were heated in chloroform for 1 hour. Colorless crystals separated from the cool solution after a day. The benzyl groups on tin has been cleaved in the reaction. In the previous study, a heating time of 3 hours gave the pentabromidochloridostannate (Jang *et al.*, 2009).

### Refinement

Hydrogen atoms were placed at calculated positions (C–H 0.95–0.98, N–H 0.88 Å) and were treated as riding on their parent atoms, with *U*(H) set to 1.2–1.5 times *U*_eq_(C,*N*).

The three halogen atoms in the stannate are disordered. The sum of the occupancies of the three bromide atoms refined to nearly 2.33Br and 0.67Cl atoms; the total occupancy of the disordered bromide atoms was then fixed as exactly 2.333. The occupancy of the disordered chloride atoms was similarly set to be 0.667. The anisotropic displacement parameters of each pair of Br/Cl atoms were restrained to be identical.

### Crystal data

**Table:**

(C_7_H_11_N_2_)_2_[SnBr_4.67_Cl_1.33_]	*F*(000) = 740
*M**_r_* = 785.15	*D*_x_ = 2.277 Mg m^−^^3^
Monoclinic, *P*2_1_/*c*	Mo *K*α radiation, λ = 0.71073 Å
Hall symbol: -P 2ybc	Cell parameters from 4263 reflections
*a* = 8.4530 (2) Å	θ = 2.5–28.3°
*b* = 11.9036 (2) Å	µ = 9.42 mm^−^^1^
*c* = 11.9093 (2) Å	*T* = 100 K
β = 107.109 (1)°	Irregular block, colorless
*V* = 1145.30 (4) Å^3^	0.30 × 0.25 × 0.20 mm
*Z* = 2	

### Data collection

**Table:**

Bruker SMART APEX diffractometer	2622 independent reflections
Radiation source: fine-focus sealed tube	2240 reflections with *I* > 2σ(*I*)
graphite	*R*_int_ = 0.033
ω scans	θ_max_ = 27.5°, θ_min_ = 2.5°
Absorption correction: multi-scan (*SADABS*; Sheldrick, 1996)	*h* = −10→10
*T*_min_ = 0.504, *T*_max_ = 0.746	*k* = −15→15
10319 measured reflections	*l* = −14→15

### Refinement

**Table:**

Refinement on *F*^2^	Primary atom site location: structure-invariant direct methods
Least-squares matrix: full	Secondary atom site location: difference Fourier map
*R*[*F*^2^ > 2σ(*F*^2^)] = 0.023	Hydrogen site location: inferred from neighbouring sites
*wR*(*F*^2^) = 0.060	H atoms treated by a mixture of independent and constrained refinement
*S* = 0.99	*w* = 1/[σ^2^(*F*_o_^2^) + (0.0343*P*)^2^ + 0.6005*P*] where *P* = (*F*_o_^2^ + 2*F*_c_^2^)/3
2622 reflections	(Δ/σ)_max_ = 0.001
127 parameters	Δρ_max_ = 0.80 e Å^−^^3^
6 restraints	Δρ_min_ = −0.87 e Å^−^^3^

### Fractional atomic coordinates and isotropic or equivalent isotropic displacement parameters (Å^2^)

**Table:**

	*x*	*y*	*z*	*U*_iso_*/*U*_eq_	Occ. (<1)
Sn1	0.5000	0.5000	0.5000	0.01384 (8)	
Br1	0.50914 (5)	0.63592 (3)	0.66901 (3)	0.02247 (12)	0.6143 (14)
Br2	0.58481 (5)	0.33875 (3)	0.64866 (3)	0.02405 (12)	0.8309 (9)
Br3	0.80683 (4)	0.53911 (3)	0.52227 (3)	0.02760 (12)	0.8878 (10)
Cl1	0.50914 (5)	0.63592 (3)	0.66901 (3)	0.02247 (12)	0.3858 (14)
Cl2	0.58481 (5)	0.33875 (3)	0.64866 (3)	0.02405 (12)	0.1122 (10)
Cl3	0.80683 (4)	0.53911 (3)	0.52227 (3)	0.02760 (12)	0.1691 (9)
N1	0.6521 (4)	0.8743 (2)	0.5886 (3)	0.0309 (7)	
H1	0.598 (5)	0.812 (2)	0.593 (4)	0.061 (14)*	
N2	0.9135 (3)	1.1561 (2)	0.5550 (2)	0.0231 (6)	
C1	0.7281 (4)	0.9350 (3)	0.6844 (3)	0.0304 (8)	
H1A	0.7212	0.9116	0.7590	0.036*	
C2	0.8143 (4)	1.0288 (3)	0.6765 (3)	0.0259 (7)	
H2	0.8683	1.0696	0.7457	0.031*	
C3	0.8251 (4)	1.0670 (3)	0.5661 (3)	0.0190 (6)	
C4	0.7363 (4)	1.0019 (3)	0.4663 (3)	0.0228 (7)	
H4	0.7345	1.0249	0.3896	0.027*	
C5	0.6553 (4)	0.9080 (3)	0.4810 (3)	0.0297 (8)	
H5	0.5994	0.8645	0.4142	0.036*	
C6	1.0103 (4)	1.2201 (3)	0.6573 (3)	0.0349 (8)	
H6A	0.9356	1.2652	0.6883	0.052*	
H6B	1.0876	1.2697	0.6340	0.052*	
H6C	1.0725	1.1680	0.7182	0.052*	
C7	0.9124 (4)	1.1989 (3)	0.4397 (3)	0.0293 (7)	
H7A	0.9577	1.1418	0.3985	0.044*	
H7B	0.9802	1.2670	0.4496	0.044*	
H7C	0.7985	1.2166	0.3937	0.044*	

### Atomic displacement parameters (Å^2^)

**Table:**

	*U*^11^	*U*^22^	*U*^33^	*U*^12^	*U*^13^	*U*^23^
Sn1	0.01350 (14)	0.01560 (14)	0.01208 (14)	−0.00189 (10)	0.00326 (11)	−0.00020 (11)
Br1	0.0329 (2)	0.0187 (2)	0.0178 (2)	−0.00322 (16)	0.01055 (17)	−0.00423 (15)
Br2	0.0329 (2)	0.01902 (19)	0.01671 (19)	−0.00065 (14)	0.00180 (15)	0.00479 (13)
Br3	0.01394 (18)	0.0409 (2)	0.0279 (2)	−0.00705 (13)	0.00609 (14)	−0.00114 (15)
Cl1	0.0329 (2)	0.0187 (2)	0.0178 (2)	−0.00322 (16)	0.01055 (17)	−0.00423 (15)
Cl2	0.0329 (2)	0.01902 (19)	0.01671 (19)	−0.00065 (14)	0.00180 (15)	0.00479 (13)
Cl3	0.01394 (18)	0.0409 (2)	0.0279 (2)	−0.00705 (13)	0.00609 (14)	−0.00114 (15)
N1	0.0277 (16)	0.0246 (15)	0.0427 (19)	0.0002 (12)	0.0141 (14)	0.0088 (14)
N2	0.0235 (14)	0.0244 (14)	0.0195 (14)	−0.0029 (11)	0.0032 (11)	−0.0017 (11)
C1	0.0298 (19)	0.037 (2)	0.0273 (18)	0.0121 (15)	0.0138 (15)	0.0114 (16)
C2	0.0256 (17)	0.0335 (18)	0.0190 (17)	0.0045 (14)	0.0071 (14)	0.0010 (13)
C3	0.0157 (14)	0.0217 (15)	0.0185 (15)	0.0046 (11)	0.0036 (12)	0.0009 (12)
C4	0.0214 (15)	0.0252 (16)	0.0198 (16)	−0.0003 (13)	0.0030 (13)	−0.0026 (13)
C5	0.0238 (17)	0.0282 (18)	0.033 (2)	−0.0001 (13)	0.0021 (15)	−0.0037 (15)
C6	0.033 (2)	0.035 (2)	0.033 (2)	−0.0101 (15)	0.0035 (16)	−0.0085 (16)
C7	0.0300 (18)	0.0285 (18)	0.0270 (18)	−0.0048 (14)	0.0045 (14)	0.0082 (14)

### Geometric parameters (Å, °)

**Table:**

Sn1—Br1	2.5658 (4)	C1—C2	1.351 (5)
Sn1—Cl1^i^	2.5658 (4)	C1—H1A	0.9500
Sn1—Br1^i^	2.5658 (4)	C2—C3	1.419 (4)
Sn1—Br2	2.5663 (3)	C2—H2	0.9500
Sn1—Cl2^i^	2.5663 (3)	C3—C4	1.433 (4)
Sn1—Br2^i^	2.5663 (3)	C4—C5	1.349 (5)
Sn1—Cl3^i^	2.5709 (3)	C4—H4	0.9500
Sn1—Br3^i^	2.5709 (3)	C5—H5	0.9500
Sn1—Br3	2.5709 (3)	C6—H6A	0.9800
N1—C1	1.343 (5)	C6—H6B	0.9800
N1—C5	1.351 (5)	C6—H6C	0.9800
N1—H1	0.882 (10)	C7—H7A	0.9800
N2—C3	1.327 (4)	C7—H7B	0.9800
N2—C6	1.466 (4)	C7—H7C	0.9800
N2—C7	1.462 (4)		
			
Br1—Sn1—Cl1^i^	180.0	Br3^i^—Sn1—Br3	180.000 (17)
Br1—Sn1—Br1^i^	180.0	C1—N1—C5	120.5 (3)
Cl1^i^—Sn1—Br1^i^	0.000 (14)	C1—N1—H1	122 (3)
Br1—Sn1—Br2	89.576 (13)	C5—N1—H1	118 (3)
Cl1^i^—Sn1—Br2	90.424 (13)	C3—N2—C6	121.7 (3)
Br1^i^—Sn1—Br2	90.424 (13)	C3—N2—C7	121.6 (3)
Br1—Sn1—Cl2^i^	90.424 (13)	C6—N2—C7	116.6 (3)
Cl1^i^—Sn1—Cl2^i^	89.576 (12)	N1—C1—C2	121.2 (3)
Br1^i^—Sn1—Cl2^i^	89.576 (12)	N1—C1—H1A	119.4
Br2—Sn1—Cl2^i^	180.0	C2—C1—H1A	119.4
Br1—Sn1—Br2^i^	90.424 (13)	C1—C2—C3	120.8 (3)
Cl1^i^—Sn1—Br2^i^	89.576 (12)	C1—C2—H2	119.6
Br1^i^—Sn1—Br2^i^	89.576 (12)	C3—C2—H2	119.6
Br2—Sn1—Br2^i^	180.0	N2—C3—C2	122.7 (3)
Cl2^i^—Sn1—Br2^i^	0.00 (2)	N2—C3—C4	121.5 (3)
Br1—Sn1—Cl3^i^	89.529 (12)	C2—C3—C4	115.7 (3)
Cl1^i^—Sn1—Cl3^i^	90.471 (12)	C5—C4—C3	120.2 (3)
Br1^i^—Sn1—Cl3^i^	90.471 (12)	C5—C4—H4	119.9
Br2—Sn1—Cl3^i^	90.248 (12)	C3—C4—H4	119.9
Cl2^i^—Sn1—Cl3^i^	89.752 (12)	C4—C5—N1	121.4 (3)
Br2^i^—Sn1—Cl3^i^	89.752 (12)	C4—C5—H5	119.3
Br1—Sn1—Br3^i^	89.529 (12)	N1—C5—H5	119.3
Cl1^i^—Sn1—Br3^i^	90.471 (12)	N2—C6—H6A	109.5
Br1^i^—Sn1—Br3^i^	90.471 (12)	N2—C6—H6B	109.5
Br2—Sn1—Br3^i^	90.248 (12)	H6A—C6—H6B	109.5
Cl2^i^—Sn1—Br3^i^	89.752 (12)	N2—C6—H6C	109.5
Br2^i^—Sn1—Br3^i^	89.752 (12)	H6A—C6—H6C	109.5
Cl3^i^—Sn1—Br3^i^	0.00 (2)	H6B—C6—H6C	109.5
Br1—Sn1—Br3	90.471 (12)	N2—C7—H7A	109.5
Cl1^i^—Sn1—Br3	89.529 (12)	N2—C7—H7B	109.5
Br1^i^—Sn1—Br3	89.529 (12)	H7A—C7—H7B	109.5
Br2—Sn1—Br3	89.752 (12)	N2—C7—H7C	109.5
Cl2^i^—Sn1—Br3	90.248 (12)	H7A—C7—H7C	109.5
Br2^i^—Sn1—Br3	90.248 (12)	H7B—C7—H7C	109.5
Cl3^i^—Sn1—Br3	180.000 (17)		
			
C5—N1—C1—C2	−2.3 (5)	C1—C2—C3—N2	−177.4 (3)
N1—C1—C2—C3	0.9 (5)	C1—C2—C3—C4	1.5 (5)
C6—N2—C3—C2	1.6 (5)	N2—C3—C4—C5	176.1 (3)
C7—N2—C3—C2	−175.2 (3)	C2—C3—C4—C5	−2.8 (5)
C6—N2—C3—C4	−177.3 (3)	C3—C4—C5—N1	1.6 (5)
C7—N2—C3—C4	5.9 (5)	C1—N1—C5—C4	1.0 (5)

Symmetry codes: (i) −*x*+1, −*y*+1, −*z*+1.

### Hydrogen-bond geometry (Å, °)

**Table:**

*D*—H···*A*	*D*—H	H···*A*	*D*···*A*	*D*—H···*A*
N1—H1···Br1	0.88 (1)	2.48 (2)	3.334 (3)	162 (4)
